# An Investigation of a Frontal Negative Slow Wave in a Virtual Hedonic Purchase Task

**DOI:** 10.3389/fnhum.2021.674312

**Published:** 2021-06-24

**Authors:** Yupeng Mei, Kunpeng Jing, Lele Chen, Rui Shi, Zhijie Song

**Affiliations:** School of Economics and Management, Yanshan University, Qinhuangdao, China

**Keywords:** frontal negative slow-wave, inhibition, impulsivity, N2, consumer neuroscience

## Abstract

There is a connection between the frontal negative slow wave (FNSW) and the arousal inhibition in the hedonic purchase context. To calculate the FNSW (400–800 ms), event-related potentials (ERPs) method was applied to depict the neural substrates on prudent and impulsive consumers’ behaviors within various states of promotion. Promotion types include the pure price promotion and the mixed promotion (a mixture of a charitable donation and a discount). Behaviorally, consumers response more quickly in the pure price promotion condition and they express a preference for the mixed promotion. More importantly, a larger FNSW emerged in the impulsive consumers than the prudent, suggesting that the former might tend to control their eagerness to consume hedonic items. Compared with the price promotion as the worse option, the mixed promotion as the better option caused more perceptual conflict, leading to an increase in N2 amplitude. It suggests that consumers incline to reject the worse offers. These results also reveal that people primarily have to search negative promotion information by their insight and subsequently impulsive consumers inhibit the responses to the promotion information. The method of ERPs and FNSW should be helpful for marketing researchers and professionals on hedonic consumption and sales promotion.

## Introduction

Over the past few years, the frontal negative slow wave (FNSW) has been discussed in the Concealed Information Test (CIT) as well as consumer neuroscience research. The CIT is used for memory detection research (Verschuere et al., 2011) and is mainly done to investigate whether a perpetrator knows crime-relevant information by using physiological responses. During the CIT, examinees are presented a crime-relevant item and several crime-irrelevant items. Innocent persons cannot differ a crime-relevant item from crime-irrelevant items whereas suspects can recognize crime-relevant information and try to pretend to make the same response as the former group. However, for examinees with involvement in the crime, the change in physiological responses to crime-relevant item can be observed, such as an increase in skin conductance, reduction in heart rate, and suppression of respiration (Gamer, 2011; Matsuda and Nittono, 2018b). Furthermore, the FNSW has been found in the CIT research. When subjects were instructed as a perpetrator, they would select to inhibit experienced physiological arousal for crime-relevant item in order not to be detected, manifested in an increase in FNSW (Matsuda and Nittono, 2015b, 2018a). In addition, the source estimation with Standardized Low Resolution Brain Electromagnetic Tomography (sLORETA) for FNSW showed that greater activation (crime-relevant – crime-irrelevant items) emerged in the right middle frontal gyrus and right inferior rate frontal gyrus when subjects attempted to conceal crime-irrelevant item (Matsuda and Nittono, 2018a).

Different from reflecting a specific process to concealment, a FNSW may be relevant to consumer preference (Goto et al., 2017). In a virtual shopping task, participants were exposed to a product picture, subsequently answered questions pertaining to their liking for the product, and finally decided whether to purchase the product or not with its price. According to participant’s liking, products were categorized as low preference, middle preference and high preference. Event-related potentials (ERPs) were measured when a product picture was presented. Compared with other products, motivational engagement toward highly preferred products was increased, leading to a discrepancy in FNSW (400–800 ms) (Goto et al., 2017).

Considering that the FNSW is associated with arousal inhibition (i.e., inhibiting the response to crime-relevant item) (Matsuda and Nittono, 2015b, 2018a), it is noted whether the FNSW (400–800 ms) related to preference for consumer goods reflects a process of inhibiting responses to some information as well in some shopping context. Wertenbroch (1998) showed that, in impulsive consumers, the expected cost of consuming more hedonic items was less than utilitarian items. In contrast, prudent people displayed a reversed conduct that bolsters the assertion that the mindset of hedonics is different, and it also portrays an inhibition of desire to use hedonic products for fun in impulsive consumers (Wertenbroch, 1998). Thus, it is worthy exploring whether different hedonic purchase behaviors between impulsive and prudent consumers can be manifested in a discrepancy in FNSW (400–800 ms) reflecting inhibition.

To rule out the explanation that the discrepancy in FNSW between impulsive and prudent consumers is a result of differential willingness to purchase in association with consumer preference (Goto et al., 2017), we also investigate the effect of two promotion types, price promotion and a mixture of promotion (a combination of a discount and a charitable donation), on hedonic purchase decision-making. In daily life, hedonic products are unnecessary to our basic well-being. As a result, consumers need good reasons to justify hedonic consumption. Price promotion (Kivetz and Zheng, 2017), donation to charity (Strahilevitz and Myers, 1998), gift giving (Lu et al., 2016) can be served as justifications. For instance, price promotion could ease the conflict between the desire for indulgence and the fact that the hedonic purchase is insignificant for their daily life and, as such, drives consumers to better achieve their hedonic goals (Kivetz and Zheng, 2017). Donation to charity has the same positive effect on purchase rate for hedonic products (Strahilevitz and Myers, 1998). Moreover, monetary incentives have either positive or negative influence in donation to charity. For instance, Landry et al. (2010) find that monetary incentives crowd out charitable donations from prior donors, but not from new ones. However, monetary reward can increase donation levels in blood drives (Lacetera and Macis, 2010). Though price promotion is a kind of monetary incentive and as such, exerts an effect on donation to charity, it remains unclear whether hedonic products with both a discount and a charitable donation are a better or worse purchase choice than those only with price promotion.

To improve the justification of the current study, we also have insight into neural mechanisms underlying hedonic purchase decision-making in various promotions. Specifically, we discussed N2 component in the present study other than the FNSW (400–800 ms). N2 as a common negative-going ERP component peaks around 250–350 ms after stimulus presentation with a maximum over the frontal area (Folstein and Van Petten, 2008). In consumer neuroscience research, N2 is related to perceptual conflict (e.g., Ma et al., 2007; Wang et al., 2016). For example, when participants were informed that the luxury products were counterfeit, they expected that these items would be with conspicuous brand logo. Thus, if brand logo for counterfeit product is inconspicuous, greater anticipation conflict would be induced and increased N2 amplitude would emerge (Zhang et al., 2019). In the present study, the effect of easing the intra-personal conflict that hedonic consumption is unnecessary for basic needs is different between the mixed promotion and the pure price promotion, which might lead to a difference in N2 amplitude.

In summary, we predict that a larger FNSW would emerge in impulsive consumers who inhibit the desire for hedonic products and the response to promotion information compared with prudent consumers. We expect that purchase behaviors in the mixed and pure price promotions could be manifested in N2 component since these promotions could have differential influence in relieving intra-personal conflict.

## Materials and Methods

### Participants

A prior power analysis suggested a sample size of 33 participants to detect medium-sized effects in a mixed ANOVA design (*f* = 0.25, α = 0.05, β = 0.80; G^∗^Power 3.1; Faul et al., 2009). In order to retain the size at least, the experiment was completed by forty undergraduates (15 males, mean age: 19.40; 25 females, mean age: 19.28) who had normal or corrected-to-normal vision with no history of neurological disorders or mental diseases. All participants reported by themselves that they were right-handed, and they were all Chinese native speakers. This study was approved by the institutional review board and written consent was provided before the experiment.

### Experimental Stimuli

**FIGURE 1:**
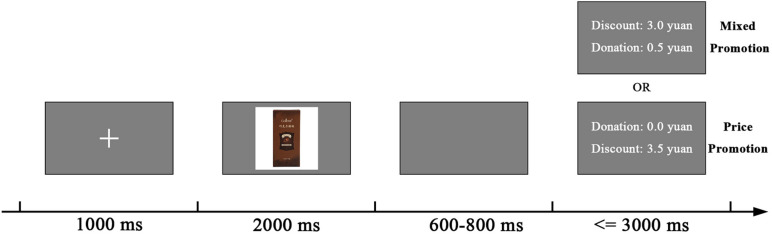
Sequence of stimuli in each trial. Epochs are extracted after promotion information onset.

A total of (50) images of snacks (i.e., a small meal, like a chocolate bar) as hedonic items whose hedonic score is higher than utilitarian score (Jing et al., 2019) were chosen and manipulated to a similar size, 300 × 300 pixels. As shown in Figure 1, in order to have enough trials and acceptable signal to noise ratio, the following amounts of discount, 1.5, 2.5, 3.5, 4.5, or 5.5 yuan, and 0 yuan donation was considered as pure price promotion, while 1, 2, 3, 4, or 5 yuan discount and a fixed 0.5 yuan donation was done as a mixed promotion.

### Procedures

All the items (totaling nine) from impulsivity scale validated by Rook and Fisher (1995) were decided to measure consumer’s impulsivity. In order to give the Chinese version of these items, we followed the Brislin’s (1980) translation/translation-back procedure. Two Chinese doctoral candidates majoring in consumer behavior translated the original items into Chinese, respectively. The two translators and the authors discussed these two versions together to develop the initial questionnaire which was subsequently translated into English by two English-speaking translators having no knowledge of the consumer’s impulsivity. And two professors of consumer behavior and other people mentioned above reviewed all translations for logical consistencies, contextual relevance and clarity to reach consensus. Finally, the scale was modified again to become clearer and more understandable based on a pilot survey of 15 consumers for feedback on these items, and it was a nine-point scale (1 = strongly disagree, 9 = strongly agree). Consistent with other hedonic consumption research (e.g., Okada, 2005; Ramanathan and Williams, 2007), a median split categorized the participants as either hedonics (*N* = 20, higher than median) or prudents (*N* = 20, lower than median) by the impulsivity scale (α = 0.91; median = 33.5). To reduce the demand effect the scale may have on the subject responses, these items were completed 1 month before the experiment.

Participants sat in a comfortable chair 80 cm away from the 23-inch monitor (1,360 × 768 pixels, 60 Hz). The Psychophysics Toolbox (Brainard, 1997) was used to present the stimuli. As shown in Figure 1, the background color was gray during the experiment and subjects were firstly exposed to a fixated cross for 1,000 ms preceding a snack picture for 2,000 ms. After a 600–800 ms empty screen, promotion information was presented including two lines, one of which was “Donation: 0.5 (or 0.0) yuan” or “Discount: 1.0 (2.0, 3.0, 4.0, 5.0) (or 1.5, 2.5, 3.5, 4.5, 5.5) yuan.” Meantime, participants were instructed to decide whether to purchase this snack with this offer within 3000 ms. Pressing “f” and “j” meant “buy” and “not buy,” respectively, for half of the participants and the opposite pattern was for the others. The relative position of donation and discount conditions was counterbalanced. The discount depth difference remained 0.5 yuan for the identical picture, and each discount was assigned to each picture at random, but the number of promotion information of each discount was equal. 100 trials in total with two blocks were pseudorandomized and all promotion conditions for each product did not appear on two consecutive trials of each block.

Before the experiment, subjects were informed that the original price of all products was 10 yuan (approximately 1.42 dollars). Each time they made purchase decision, participants had a virtual allocation of 30 yuan (approximately 4.25 dollars). To better simulate shopping context, we would select a product at random from the items with an offer participant had purchased during the experiment to “sell” them. Subjects at last could receive this product and “cash savings” (30 yuan minus the present price of the product), and for the mixed promotion, they would also donate 0.5 yuan to the Project Hope that is a familiar good cause in China and plans to help youngsters in disadvantaged areas. Following Schaefer et al. (2016) research, a punitive measure was actualized for potential inclinations that subjects could just purchase few items. On the off chance that the quantity of the conditions where participants would purchase hedonic items was <10, they would lose 9 yuan. If the number was somewhere in the range of 10 and 12, they would lose 6 yuan and if somewhere in the range of 13 and 15, 3 yuan was lost. Participants would not lose any cash if the amount was >15.

### Behavioral Recordings and Analysis

Behavioral recordings were created separately for four experimental conditions in a 2 (promotion type: mixed vs. pure, a within-subject factor) × 2 (impulsivity: impulsive vs. prudent, a between-subject factor) mixed design. Purchase rate and reaction time were recorded using the Psychophysics Toolbox (Brainard, 1997). Each subject’s purchase rate referred to a proportion of items selected to purchase. Reaction time referred to a time segment from timing of presenting promotion information to that of making final decision. Repeated-measured analyses of variance (ANOVAs) were performed for behavioral data.

### EEG Recording and Analysis

Each subject’s scalp EEG was recorded using a Brain actiCHamp amplifier (Brain Products GmbH, Munich, Germany) and from 64 Ag/AgCl electrodes with a sampling rate of 500 Hz in a cap. Impedance was kept below 10 kΩ and data were filtered online at 0.05–100 Hz band-passes. Recordings were referenced online to the Cz site and the mean of the left and right mastoids served as an offline reference. The electrodes placed supra- and intra-orbital to both eyes and lateral to the outer canthi of both eyes were used to measure the electrooculogram. BrainVision Analyzer 2.1 (Brain Products) was selected to preprocess the EEG data filtered offline with a low-pass filter at 30 Hz and epochs were extracted between 200 ms before as a baseline and 800 ms after promotion information onset. Epochs with a deflection exceeding ±100 μV were rejected and eye movements were corrected using the algorithm of Gratton et al. (1983).

On the basis of the grand average waveforms and some literatures on consumer neuroscience (Wang et al., 2016; Goto et al., 2017; Jing et al., 2019), F3, Fz, and F4, as a frontal cluster, were selected for the N2, measured by the mean amplitude of the 270–330 ms time window, respectively. Similarly, the time window of 400–800 ms was analyzed for the FNSW including F3, Fz, and F4, as a frontal cluster (Matsuda and Nittono, 2015b, 2018a). Repeated-measured analyses of variance (ANOVAs) were performed for ERP data. Spearman correlation analyses were conducted between the N2 amplitude and the reaction time as well as the N2 amplitude and the purchase rate.

## Results

### Behavioral Results

**FIGURE 2:**
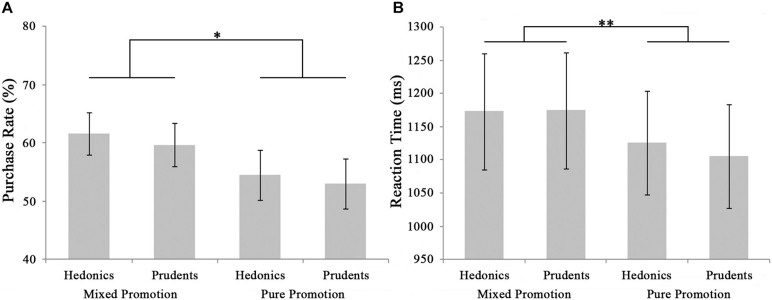
Behavioral results. The purchase rate **(A)** and reaction time **(B)** for every condition, and the purchase rate of each discount amount in the mixed promotion and pure promotion conditions, respectively. The error bars suggest standard error of the mean. ^∗^*p* < 0.05, ^∗∗^*p* < 0.01.

The ANOVA analysis of 2 (promotion type: pure vs. mixed, a within-subjects factor) × 2 (consumer trait: impulsive vs. prudent consumers, a between-subjects factor) was conducted for the purchase rate and the reaction time, respectively. The main effect of promotion type was significant in the purchase rate [*F*_(__1_,_38__)_ = 4.290, *p* = 0.045, $\eta_{p}^{2}$ = 0.101] as well as the time of reaction [*F*_(__1_,_38__)_ = 8.725, *p* = 0.005, $\eta_{p}^{2}$ = 0.187]. As portrayed in Figure 2, the purchase rate in the mixed promotion condition (Mean = 0.607, SE = 0.026) was higher than that in the pure promotion condition (Mean = 0.537, SE = 0.030). In contrast, the reaction time in the mixed promotion condition (Mean = 1173.670 ms, SE = 62.144) was longer than that in the pure promotion condition (Mean = 1115.670 ms, SE = 55.283). The other effects for the purchase rate (*F*s < 0.14, *p*s > 0.71) or reaction time (*F*s < 0.30, *p*s > 0.58) lacked significance.

### ERP Results

**FIGURE 3:**
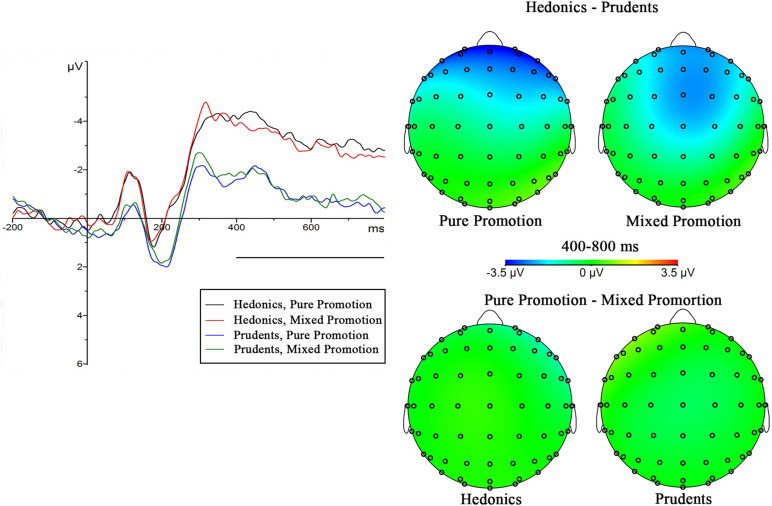
The grand-averaged ERP waveforms (left) for every condition collected over electrodes F3, Fz, and F4. The difference maps (400–800 ms) were in the right.

N2 and FNSW were, respectively, collected in a 2 (promotion type) × 2 (consumer trait) ANOVA. The ANOVA of N2 revealed a significant main effect on the promotion type [*F*_(__1_,_38__)_ = 4.467, *p* = 0.041, $\eta_{p}^{2}$ = 0.105], however, not in consumer trait [*F*_(__1_,_38__)_ = 2.636, *p* = 0.113]. Figure 3 indicated that a larger N2 amplitude in the mixed promotion (Mean = −3.148 μV, SE = 0.490) was found than in the pure promotion (Mean = −2.709 μV, SE = 0.509). The effect of interaction between promotion type and was without significance [*F*_(__1_,_38__)_ = 0.008, *p* = 0.928]. Spearman correlation analyses showed that the N2 amplitude on F4 (*r* = −0.266, *p* < 0.05) was negatively related to the reaction time and on F3 (*r* = 0.248, *p* < 0.05) was positively done to the purchase rate.

The FNSW result produced a significant main effect of consumer trait [*F*_(__1_,_38__)_ = 4.163, *p* = 0.048, $\eta_{p}^{2}$ = 0.099] that is different from the promotion type [*F*_(__1_,_38__)_ = 0.282, *p* = 0.598]. As depicted in Figure 3, in impulsive consumers (Mean = −3.225 μV, SE = 0.765) revealed a higher FNSW than in the prudent (Mean = −1.017 μV, SE = 0.765). The interaction effect for the promotion type and indulgence came out without significance [*F*_(__1_,_38__)_ = 0.589, *p* = 0.448].

## Discussion

The core essence of this study is to explore FNSW which emerges in consumers during their purchase of hedonic items apart from the CIT and to reveal psychological process relevant to the FNSW. Moreover, we also consider purchase rate, reaction time and N2 component to increase the justification of explanation for FNSW in the present study.

As expected, a higher FNSW amplitude was evoked in the impulsive people than in the prudent. Since the FNSW reflects an inhibition of responses to crime-relevant information (Matsuda and Nittono, 2015b, 2018a), one could speculate that the FNSW (400–800 ms) is not only related to consumer preference (Goto et al., 2017), but also it might reflect a process of inhibiting the response to some information in the context of purchase decision-making. Promotion as a common marketing strategy could effectively spur the demand for hedonic products (Kivetz and Zheng, 2017). In this experiment, when exposed to various promotion information, impulsive consumers tended to control their eagerness to buy hedonic items (Wertenbroch, 1998) and they selected to inhibit the response to promotion information, leading to an increase in FNSW compared with prudent consumers. In the CIT, subjects are instructed to try to keep the same brain activity for crime-relevant items as other items. However, the present experiment did not require participants to deliberately inhibit their desire and response. The FNSW in this study might reflect the inhibition built upon spontaneous and elaborative process.

As a complementary finding, we observed a significantly higher purchase rate in the mixed promotion (a combination of a discount and a charitable donation) than in the pure price promotion. Although donation to charity has the same effect in promoting hedonic purchase as price promotion, the positive synergy between price promotion and donation-based promotion was found, and consumers preferred hedonic products with combined discount and donation offer. In addition, there was no significant main effect of impulsivity and interaction effect for purchase rate. The influence for two promotion types in easing the intra-person conflict that hedonic consumption did not fill basic needs might be consistent across impulsive and prudent consumers. As preferences for promotions are not significantly different between these two groups of consumers, these results also provide indirect evidence to rule out the alternative explanation that the difference in FNSW is a result of subjective preference.

As for the reaction time, we obtained a longer reaction time in the mixed promotion. Reaction time is associated with task difficulty and cognitive load (Sweller, 1988; Cowen et al., 2002). Consumers made easily final decision in the mixed promotion condition whereas they exerted extra cognitive effort in the price promotion condition. We contend that the reason for responses more quickly to the price promotion than the mixed promotion is that the decision pattern during the experiment is how to not purchase. Because purchase intention is higher in the mixed promotion condition, it is more difficult to search negative information to reject purchase. As a matter of fact, the N2 results can support this argument.

Our examination gained us a significant main effect of promotion type for the N2 component. Consumer neuroscience studies indicated that the component is positively related to perceptual conflict (e.g., Wang et al., 2016; Jing et al., 2019). The result for N2 has provided the argument about reaction time with evidence. As purchase rate’s results showed, consumers have a preference for the mixture of a discount and a charitable donation. Although the mixed promotion has a more positive influence in relieving the conflict that hedonic consumption is unnecessary for our daily life, the decision pattern is to reject hedonic products with some offer. Therefore, during this experiment, participants anticipated selecting the pure price promotion as a rejected option. When the mixed promotion was presented, anticipation conflict was elicited and thus an increased N2 amplitude emerged. Moreover, that N2 component reflected promotion information processing provides neurophysiological evidence for the explanation of FNSW in hedonic purchase context. Specifically, though we contend that the FNSW is relevant to the inhibition of desire for hedonic consumption, one alternative explanation is that compared with prudent consumers, impulsive consumers have an increase in motivational engagement with promotion information and thus a higher FNSW emerges. However, the N2 result showed that participants were motivated to reject products with price promotion offer, that is, motivational engagement with purchase based on promotions can be reflected in N2 component. In contrast, the consumer preference for promotions did not reflect in the FNSW (insignificant main effect of promotion type). Thus, the FNSW in the current study is specific to a process of motivational engagement with inhibition rather than consumption preference.

N2 and FNSW components indicate a two-stage pattern. Firstly, regardless of impulsive and prudent consumers, they need to depend on perception, experiences and understanding when they conclude whether to purchase hedonic items or not over the span of their regular day to day existence. As the N2 result shows, the decision pattern of how to not purchase drives consumers to anticipate the worse offer corresponding to products. After they indicate a preference, impulsive consumers have to inhibit physiological responses to promotion information, but it is not the case in prudent consumers, as the FNSW result shows. The pattern is similar to the psychological constructs of behavioral activation system (BAS) and behavioral inhibition system (BIS). The BAS reflects approach to positive stimuli and is sensitive to signals of reward and non-punishment, and the BIS is sensitive to signals of punishment and non-reward and inhibits behavior which may lead to negative outcomes (Gray, 1981, 1982). Promotion type and impulsivity might be related to BAS and BIS, respectively. When promotion information was presented, the BAS was firstly activated. Consumers’ goal was to reject the worse offer. Thus, greater activation in the BAS in the pure price promotion condition drives subjects to increase movement toward the goal. Secondly, activity in the BIS causes impulsive consumers inhibition of movement toward the goal, that is, to inhibit the responses to promotion information.

## Implications

Event-related potentials as a non-invasive technology can provide a window into consumer’s brain activity and thus produce a different interpretation of consumer behaviors from using behavioral approaches. Participants express a preference for the mixed promotion regardless of impulsivity, at the same time, they anticipate rejecting the pure promotion. However, impulsive consumers produce a larger FNSW and have an inhibition of purchase desire, which makes it difficult to associate with the current behavioral results of purchase rate and reaction time. The difference of N2 component between promotion types helps marketing researchers to better understand consumers’ attitudes toward purchases. Researchers should also focus on the implication of FNSW only being obtained from the method of ERPs. Arousal inhibition that the FNSW represents is sometimes undetectable in hedonic purchase contexts especially when the inhibition does not determine final decisions making. Hedonic consumption brings on sensual pleasure, fantasy, fun, and feelings of guilt (Strahilevitz and Myers, 1998), and impulsive consumers have to repress their positive and negative emotions such that they can consume hedonic items frequently in the future. As the emotional experience of consumer can boost sales (Gountas and Gountas, 2007), ERPs should be regarded as an effective tool and a complement for marketing researchers to acquire a deeper understanding of behaviors on hedonic purchase.

On the other hand, the implication of FNSW in sales promotion should be discussed further. Our results suggested that the amplitude of FNSW was not affected by promotion information. According to the benefit congruency framework of sales promotion effectiveness (Chandon et al., 2000), higher purchase rate found in mixed promotion condition denotes that the mixed promotion provides more hedonic benefits than the pure price promotion and consumers place a greater weight on hedonic benefits from products. But how can we connect promotions with the FNSW? And as the FNSW is an indulgence-specific component in hedonic purchase contexts how can sales promotion work on the FNSW? Impulsive consumers are more willing to have the slight profit constantly from hedonic consumption (Wertenbroch, 1998). Therefore the benefits of promotion strategies need to be congruent, for example, the discount is shallow, but the activity continues for a long time. When consuming hedonic items with such promotion offers, impulsive consumers have an enjoyable experience. Promotion effectiveness of hedonic purchases depends on whether sales promotion helps impulsive consumers not to inhibit their impulse and to consume with positive emotions and without negative emotions. As arousal inhibition is characterized by FNSW rather than by other data obtained from behavioral method, marketing professionals need to pay more attention to whether the FNSW can be modulated by new promotion strategies targeted at impulsive people.

## Limitations and Extensions

Matsuda and Nittono (2015a) showed that the occipital dominant LPP with a frontal negativity (FNSW) was evoked by task-relevant picture not only in the concealment condition, but also in the two-item updating condition in which subjects have to update both month and date when see the relevant information. The occipital dominant LPP might reflect an effortful and controlled processing (Matsuda and Nittono, 2015a). Thus, in the current experiment, as impulsive consumers need to inhibit their desire, they could engage in additional processing and require more cognitive effort than prudent consumers, ultimately leading to a larger FNSW. In other words, the FNSW difference between the two groups might be explained by cognitive load. It must be noted that subjects need be compelled to act in manner that would evoke an FNSW in the CIT whereas the FNSW can be from an implicit attitude toward relevant information. Impulsive consumers have habitually inhibited their initial impulse in their everyday of life and they might do the same cognitive effort as prudent consumers. The logic is similar to that of some ERP studies about training effect (e.g., Xiu et al., 2018; Pan et al., 2019). In an arithmetic calculation task, for example, subjects had to judge whether the proposed solutions to arithmetic problems were correct or not, and larger size problems elicited an increased positive slow wave amplitude, and more importantly, positive slow wave decreased after training (Núñez-Peña, 2008). In other words, by practice participants could correctly answer arithmetic problems in an effortless way. Over the years, the practice of purchasing in different conditions (similar to training) promotes people by osmosis to form an impulsive or prudent purchase habit. In the current study, consumers having one of the two habits put the same effort into hedonic purchases though their brain responses could be different. Future research should focus on learning effect or training effect to check the processes reflected by a FNSW in hedonic purchase contexts.

The motivations for concealment and disclosure could also be regarded as potential limitation. In this study, we contend that impulsive consumers tend to conceal their desire when exposed to promotion information. However, some literatures seemingly support another explanation. Because hedonic products are unnecessary for basic well-being and wasteful, consumers purchasing such items does not conform to society and they are more likely to be criticized (Baumeister, 1982; Böhm and Pfister, 1996) with a sense of guilt (Prelec and Lowenstein, 1998). Impulsive consumers feel less negative self-conscious emotion, which arises from effortful and thoughtful processing over time, such as guilty, ashamed and regretful, than do prudent consumers after hedonic consumption (Ramanathan and Williams, 2007). Criticism from society does not make impulsive consumers feel much guilty. One could speculate that impulsive consumers are not affected by social norm and tend to show their desire for hedonic purchase. Based on the current experiment, future research could explore the influence of social desirability in hedonic purchase.

As a complement to the FNSW waveform analysis, we performed the global field power (GFP) analysis to explore other temporal events associated with frontal to the sequence of stimuli (see Supplementary Material). The GFP is a measure of global brain activation calculated as the root mean-squared value of the EEG signal across all electrodes with larger values for stronger electric fields (Lehmann and Skrandies, 1980). For each promotion type (price promotion and mixed promotion), we applied a point-wise paired *t*-test comparing GFP values for impulsive vs. prudent consumers with a 10-point temporal criterion for significance. Thus, we considered the first time point (from 400 ms to 800 ms) where the *t*-test was significant (with the 0.05 alpha criterion) for at least 10 consecutive data points (i.e., 20 ms at 500 Hz digitization rate). Such consecutive data points were not found. The significant difference for the FNSW not associated with a global change in field strength indicates a local modulation of frontal responses. In the future consumer neuroscience research, the GFP analysis could help researchers to obtain more information to support and extend conclusion.

Some studies have suggested that gender is in association with impulsivity (e.g., Fu et al., 2007; Wu and Huan, 2010). For example, based on a large non-clinical sample, Grano et al. (2004) showed that impulsivity could predict an increase in number of cigarettes smoked per day in women, but it was not the case in men. We tested the gender factor in this study, but there were no significant effects (*F*s < 0.23, *p*s > 0.63). One worthwhile extension of this work might focus on other products like cigarettes and alcohol which have been often studied in impulsivity, or on other reasons to justify hedonic consumption, such as gift giving in which gender has an influence (e.g., Ashwin et al., 2013).

In this study, we used S1–S2 paradigm, which is often employed in consumer neuroscience research, to investigate the effects of promotion type and impulsivity. However, oddball paradigm has been generally used by the CIT research. Future research could employ oddball paradigm to provide more direct evidence to support the notion that FNSW reflects a process of inhibiting the responses to promotion information in hedonic purchase decision making. And we can also use oddball paradigm to explore whether other ERP component having emerged in the CIT, such as P3, could be linked with consumer behaviors.

## Conclusion

In the virtual hedonic buy task, arousal inhibition is denoted by FNSW (400–800 ms). Unlike the prudent subjects the impulsive consumers repressed their brain reactions to all the promotion types which was shown in a bigger FNSW, and a larger N2 amplitude in the mixed promotion condition suggested that consumers might go to great length to search negative promotion information to reject purchase.

## Data Availability Statement

The raw data supporting the conclusions of this article will be made available by the authors, without undue reservation.

## Ethics Statement

The studies involving human participants were reviewed and approved by the Internal Review Board of the Laboratory of Cognitive Neuroscience, Yanshan University. The participants provided their written informed consent to participate in this study.

## Author Contributions

YM, KJ, LC, and RS conceived and designed the experiment. YM, KJ, and LC performed the experiment. YM and RS analyzed the data. YM, KJ, and ZS wrote and edited the manuscript. All authors contributed to the article and approved the submitted version.

## Conflict of Interest

The authors declare that the research was conducted in the absence of any commercial or financial relationships that could be construed as a potential conflict of interest.
