# Supplementary figures and images for: An Investigation of a Frontal Negative Slow Wave in a Virtual Hedonic Purchase Task
Source: Front Hum Neurosci. 2021 Jun 24;15:674312. doi: 10.3389/fnhum.2021.674312 (PMC8264297; doi:10.3389/fnhum.2021.674312)

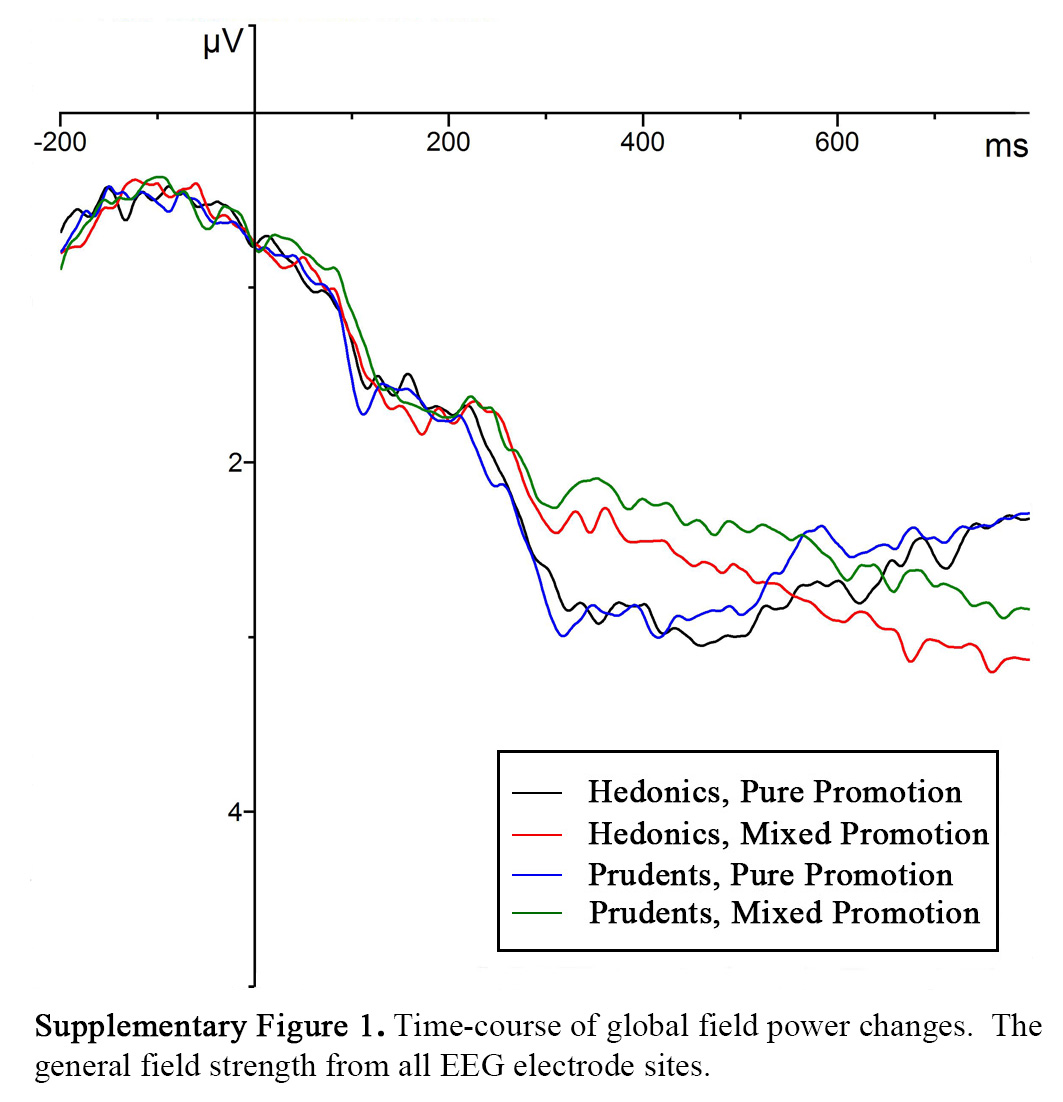

Supplement: Supplementary file 1 [file Image_1.JPEG]
